# Reciprocal facilitation between mental and visuomotor rotations

**DOI:** 10.1038/s41598-022-26397-3

**Published:** 2023-01-16

**Authors:** Jianfei Guo, Joo-Hyun Song

**Affiliations:** 1 Department of Cognitive, Linguistic and Psychological Sciences, Brown University, Box 1821, Providence, RI 02912 USA; 2 Carney Institute for Brain Science, Brown University, Providence, RI 02912 USA

**Keywords:** Psychology, Human behaviour

## Abstract

Humans exhibit remarkably complex cognitive abilities and adaptive behavior in daily life. Cognitive operation in the "*mental workspace,*" such as mentally rotating a piece of luggage to fit into fixed trunk space, helps us maintain and manipulate information on a moment-to-moment basis. Skill acquisition in the "*sensorimotor workspace,*" such as learning a new mapping between the magnitude of new vehicle movement and wheel turn, allows us to adjust our behavior to changing environmental or internal demands to maintain appropriate motor performance. While this cognitive and sensorimotor synergy is at the root of adaptive behavior in the real world, their interplay has been understudied due to a divide-and-conquer approach. We evaluated whether a separate *domain-specific* or common *domain-general operation* drives mental and sensorimotor rotational transformations. We observed that participants improved the efficiency of mental rotation speed after the visuomotor rotation training, and their learning rate for visuomotor adaptation also improved after their mental rotation training. Such bidirectional transfer between two widely different tasks highlights the remarkable reciprocal plasticity and demonstrates a common transformation mechanism between two intertwined workspaces. Our findings urge the necessity of an explicitly integrated approach to enhance our understanding of the dynamic interdependence between cognitive and sensorimotor mechanisms.

## Introduction

Historically, the information-processing theory established a one-way information flow of acquiring perceptual input, processing the information with high-level cognition, and then planning and executing appropriate actions^1–4^. Consequently, each domain has been primarily examined in isolation. On the one hand, research on "*mental workspace*," including mental imagery, visuospatial ability, and working memory, has focused mainly on the ability to store, maintain, and manipulate information while neglecting issues of sensorimotor control, the means by which perceptual and cognitive ability is behaviorally expressed^5^. On the other hand, research on *sensorimotor behavior*, including skill acquisition, has focused on the neurophysiological, anatomical, and other implicit mechanisms that control simple motor outputs with little consideration for the roles of higher-order cognitive functions^6^. These focused approaches on well-defined isolated domains afford scientific rigor and have contributed to the development of coherent bodies of work in each subsystem replete with successful explanatory theories and a rich collection of paradigms, tasks, and analytic techniques. Nevertheless, it can lose sight of the co-dependence between cognition and action, which may be fundamental to realistic adaptive behaviors in the complex real world^7–12^.

Recent evidence has consistently shown the prevalent interplay between cognition and action^7,8,13–23^. For instance, recent studies have discovered that cognitive factors such as attention play a crucial role in developing and maintaining visuomotor skills within more complex environments^10,24–27^. Guo and Song^28^ also found that perceptual discrimination performance improved as actions became more fluent (e.g., as grasping errors decreased). Importantly, they observed that grasping training prior to discrimination enhanced subsequent perceptual sensitivity, supporting the notion of a reciprocal relation between perception and action. Extensive research also showed that prior mental practice of a motor task without overt physical activity enhances movement performance (for reviews^29,30^).

Furthermore, action-specific perception theory states that humans’ ability to act influences how they perceive the environment^31,32^. The conventional "modular" sequential approach could not explain these findings. Instead, it highlights the necessity of an explicitly integrated approach to determine how cognitive operations link with sensorimotor learning processes. Thus, in the present study, using a novel behavioral training-transfer paradigm that simultaneously taps into cognitive and visuomotor domains, we investigated whether separate *domain-specific* or common *domain-general* processing governs the transformations of rotation required in visual cognition (visual imagery) and sensorimotor (visuomotor rotation adaptation) domains.

Until recently, visuomotor rotation (VMR) adaptation has been exclusively formulated as a form of error-driven implicit sensorimotor learning. In a typical VMR task, individuals make reaching movements toward a target, and a visual perturbation is applied to the cursor (e.g., the cursor is rotated 45° counterclockwise). Individuals must learn to compensate for this perturbation by reaching the opposite direction (e.g., reaching 45° clockwise). Mazzoni and Krakauer^33^ demonstrated that explicit cognitive strategies were unconsciously overridden during the adaptation process. However, the role of explicit cognitive strategies remained a topic of debate. Taylor and Ivry^34^ demonstrated that participants could flexibly combine such instructed strategies with implicit adaptive processes to optimize performance with extensive practice. Taylor et al.^35^ also dissociated explicit and implicit contributions to the visuomotor adaptation task by asking participants to report their aiming location directly. While the specific computations underlying cognitive strategies in sensorimotor learning are poorly understood, mental rotation has been considered a possible strategic transformation involved in VMR adaptation.

Mental rotation is the ability to transform a perceptual representation of an object to accurately predict how the object would look from a different angle, such as when people try to imagine how the living room would look with the furniture rearranged. The time to make judgments about a rotated object increases near-linearly with the amount of rotation required to bring the object to align with a comparison object or with a learned spatial template. Underlying operations for mental rotation include the active maintenance of a visual configuration, its transformation, and subsequent comparison to a target image^36–42^. While mental rotation has been extensively studied within the visual imagery domain, a series of studies have shown that mental rotation can be of more general application to motor performance under transformed spatial mappings such as VMR. For example, prior studies showed that mental rotation could be facilitated by simulating manual rotation covertly, and this covert stimulation activates the motor cortex^43–45^. Neurophysiological evidence showed that VMR activates the corresponding rotation of neuronal population vectors in the motor cortex^46,47^. Previous studies also showed that processing rates of VMR and visual mental rotation, revealed by the linear relation between rotated angles and response times, were positively correlated^46–50^. In addition, McDougle and Taylor^51^ suggested that the complexity of a sensorimotor task influences whether to employ a rotation computation for VMR: when a large number of reach target locations (e.g., 12) is used, participants rely on a time-consuming parametric rotation computation, whereas when a small number of locations (e.g., 2) is used, they rely on a discrete caching of stimulus–response (i.e., a look-up table). These studies demonstrating a correlated processing rate between the two rotation tasks or parametric changes in VMR mimicking mental rotation suggest that similar rather than completely different processing constraints underlie visuomotor and visual mental rotations^46,48–50^.

However, this correlational or similarity-based evidence^47–51^ has left open the question of whether perceptual and visuomotor domains merely represent rotation similarly or whether they are tightly coupled, sharing a common mechanism. The domain*-specific hypothesis* postulates that the rotation transformation involves different neural systems depending on the perceptual or motor task. However, each dedicated brain area similarly processes rotation itself. For instance, while neurophysiological evidence showed that visuomotor rotation leads to the corresponding rotation of neuronal population vectors in the primary motor cortex (M1)^46,47^, whether M1 is involved in visual imagery rotation is still a subject of debate^39,52^. Alternatively, the *domain-general hypothesis* postulates that mental rotation, perceptual or visuomotor, involves a certain brain area jointly accessed by both the perceptual and the motor systems. Therefore, the similar processing constraints observed would be due to processing features of that common brain area.

In the present study, we reasoned that direct behavioral evidence of the involvement of a common rotation operation could be obtained by examining whether performance improvement in one process can lead to changes in the other. In other words, the *domain-general* hypothesis predicts that 1) training in the visuomotor rotation will facilitate mental rotation, particularly by improving the rotation rate, but the other motor task without rotation will not (Experiment 1); reversely, 2) mental rotation training will enhance visuomotor rotation, but other cognitive tasks without a rotation judgment will not (Experiment 2). Indeed, we observed a bidirectional transfer between the two widely different tasks. This supports the hypothesis that rotation operation across cognitive and sensorimotor domains is driven by a common *domain-general* rather than *domain-specific transformation.*

## Results

### Experiment 1: visuomotor training improves mental rotation

**Figure 1:**
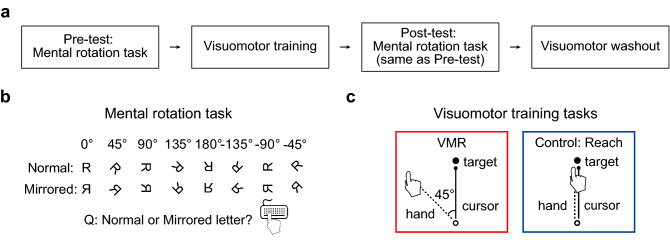
Schematics of Experiment 1. (**a**) The experimental procedure. (**b**) In the mental rotation task, participants were asked to respond whether a tilted letter (e.g., R) was a normal or a mirrored image. Four asymmetric letters (F, G, J, R) were presented as targets randomly in the tasks. (**c**) In the visuomotor training session, participants performed a visuomotor rotation (VMR; red border) task or a control reach task (blue border). In the VMR and control reach task: participants moved the cursor (small black dot) from the starting base (open circle) toward the target (big black dot), and the cursor direction (solid line) was rotated 45° clockwise from (VMR) or normally followed (control reach) the hand trajectory (dotted line).

In Experiment 1, we developed a novel training-transfer paradigm (Fig. 1a) by combining mental rotation (Fig. 1b) and visuomotor training tasks (Fig. 1c) to evaluate whether a separate *domain-specific* or common *domain-general rotational operation* drives mental and visuomotor rotation. The domain-specific hypothesis predicts no transfer across domains. In contrast, the domain-general hypothesis predicts that VMR training will facilitate the efficiency of mental rotation, but the other motor task without rotation operations will not.

To evaluate these hypotheses, all participants performed four sessions: a pre-test of mental rotation, visuomotor training, a post-test of mental rotation, and visuomotor washout (Fig. 1a). The two mental rotation sessions were identical for all participants, requiring participants to determine whether a tilted asymmetric letter (e.g., R) was a normal or a mirrored letter (Fig. 1b). Therefore, it enabled us to evaluate how mental rotation performance was affected by the different visuomotor training tasks (VMR and control reach) (Fig. 1c). The visuomotor washout session was designed to confirm that the visuomotor training effects were maintained during the post-test of mental rotation.

During the visuomotor training session, participants (n = 19) were randomly assigned to the VMR (red border) group or a control reach (blue border) group (Fig. 1c). The control reach group enabled us to estimate test–retest improvement in mental rotation and parse out contributions led by movements without rotational operation. In the *VMR task*, participants moved the cursor (small black dot) from the starting base (open circle) toward the target (big black dot). Each target direction was located in 45° increments from 0° (12 o'clock) to 315° for a total of eight possible target locations. There were two types of trials. In rotation trials, the cursor direction (solid line) was rotated 45° clockwise (CW) from the hand trajectory (dotted line). This perturbation creates movement errors, forcing the brain to learn or update new sensory-motor relationships to reestablish appropriate motor control. In no-rotation trials, the cursor direction (solid line) normally followed the hand trajectory (dotted line). Participants performed one baseline block (80 no-rotation trials) measuring inherent bias in the reaching movement toward each target and four training blocks (80 rotation trials/block). In the *control reach task*, participants moved the cursor from the starting base toward the target, and the cursor direction normally followed the hand trajectory (see “Methods: Experiment 1: Tasks and Procedures” for details).

**Figure 2:**
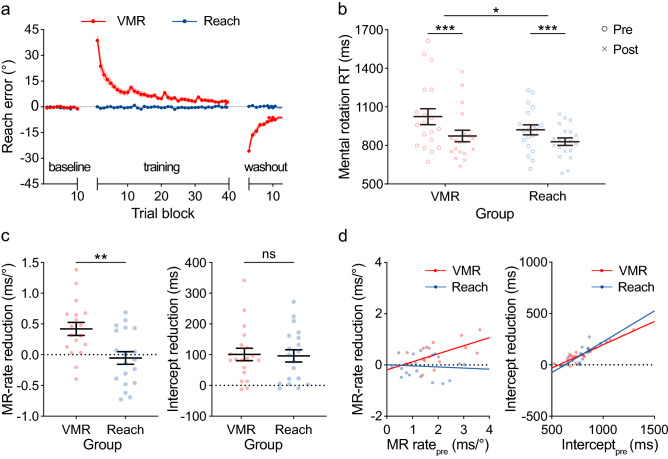
Results of Experiment 1. (**a**) Reach error of VMR (red) and control reach (blue) groups in the baseline, visuomotor training, and visuomotor washout session. (**b**) Reaction time (RT) of mental rotation tasks in the VMR (pre: 1024 ± 62.04 ms (s.e.), post: 873.9 ± 45.24 ms) and control reach group (pre: 921.8 ± 39.08 ms, post: 829.6 ± 29.50 ms). Markers represent individual participants in the pre- (circle) and post-test (cross). Black horizontal lines represent the mean with standard errors of the mean in the corresponding group. (**c**) Reduction in the mental rotation (MR) rate (left) and the intercept (right) from the pre- to post-test in the two groups. Dots represent individual participants. Larger positive numbers indicate larger reduction, i.e., performance improvement. VMR group shows significantly larger MR-rate improvement (0.42 ± 0.11 ms/°) then the reach group after the visuomotor training session (− 0.05 ± 0.10 ms/°), while their intercept improvements are not different (101.2 ± 20.20 ms vs. 96.42 ± 19.96 ms). (**d**) Relation between the pre-test performance and the improvement after visuomotor training in the MR rate (left) and intercept (right) in each participant. In terms of MR rate, less efficient participants in the pre-test improve significantly more after the visuomotor training in the VMR group (y = 0.31*x−0.19; *R*^2^ = 0.36*, p* = 0.011), while there was no difference in the control reach group (y = − 0.04*x + 0.01. *R*^2^ = 0.01*, p* > 0.250). In terms of intercept, participants with a larger intercept in the pre-test shortened their RT more in both groups after training (VMR: y = 0.45*x−257.1, *R*^2^ = 0.86*, p* < 0.001; control reach: y = 0.60*x−376.7, *R*^2^ = 0.66*, p* < 0.001). In (**a**)–(**d**), the colors of the markers and lines correspond to visuomotor training groups as depicted in Fig. 1c (red: VMR and blue: control reach). Error bars indicate standard errors of the mean. An asterisk indicates significant difference between results for the two training groups (*p < 0.05, **p < 0.01, ***p < 0.001).

As shown in Fig. 2a, we evaluated the motor performance of two groups during the visuomotor training session. In the visuomotor rotation (VMR) group (Fig. 2a: red), reach error was reduced across trial blocks in the visuomotor training (*F*(5.52, 99.43) = 40.72, *p* < 0.001, η^2^ = 0.55), suggesting enhancement in visuomotor rotation. As control, the reach task without the rotated visual feedback (Fig. 2a: blue) showed uniformly small reach error (*F*(6.60, 118.7) = 0.75, *p* > 0.25).

Then, we evaluated whether the overall mental rotation performance was differentially affected by VMR task training compared to the control reach task. To confirm that both training groups performed the mental rotation task reasonably well, we first calculated the mean accuracy of the mental rotation task separately in the pre-and post-test sessions across the VMR (pre vs. post-test: 88.9% ± 1.4% s.e., vs. 90.2% ± 1.1%) and control reach group (88.5% ± 1.2% vs. 91.4% ± 1.2%). We confirmed that the accuracy of the pre-test between the two groups was equated by an independent t-test (*t*(36) = 0.21, *p* > 0.250). In a two-way repeated measures ANOVA, participants in both groups showed higher accuracy in the post-test compared to the pre-test session (*F*(1, 36) = 11.02, *p* = 0.002, η^2^ = 0.04). There was no interaction effect (*F*(1, 36) = 1.76, *p* = 0.193). Therefore, the accuracy was equated across two groups.

We shifted our focus to the mean reaction times of the mental rotation task in the pre-and post-test sessions (Fig. 2b) and an RT difference between the two test sessions. A larger RT reduction indicates an overall larger mental rotation performance improvement. As shown in Fig. 2b, participants in both groups performed the mental rotation task significantly faster in the post-test compared to the pre-test session (*F*(1, 36) = 74.31, *p* < 0.001, η^2^ = 0.09), while the RT of the pre-test between the two groups was equated (*t*(36) = 1.40, *p* = 0.172). More importantly, the VMR group (red) showed significantly greater mental rotation improvement than the control reach group (blue), leading to a significant interaction effect (*F*(1, 36) = 4.24, *p* = 0.047, η^2^ = 0.005).

While demonstrating an improvement in mental rotation performance by VMR training, of critical interest is what specific processes were indeed improved. According to previous studies, the mental rotation process involves several steps^42,53–55^. For instance, the regression data between rotation angles and RTs were commonly used to indicate different mental rotation steps^56,57^: the slope represents the mental rotation rate (i.e., RT increment per rotation angle), and the intercept represents other processes, including object encoding, comparison, and response. Therefore, to separate the efficiency of transformation of spatial information (i.e., slope change) and other peripheral processes (i.e., intercept change), we performed linear regressions on RTs across letter rotation angles for each individual in pre- and post-test separately for both VMR (sFig. 1a, left in Supplementary material) and control reach (sFig. 1a, right) groups.

In each participant, we obtained the regression slopes across the pre- and post-test (sFig. 1b, left): VMR (pre vs. post-test: 2.40 ± 0.39 ms/° (s.e.) vs. 1.79 ± 0.30 ms/°) and control reach (1.42 ± 0.18 ms/° vs. 1.47 ± 0.22 ms/°). Note that all participants showed positive slopes during the pre-test in the VMR (range: 0.73 ~ 3.63 ms/°), *t*(18) = 6.16, *p* < 0.001, *d* = 1.41 and the control reach group (0.20–3.03 ms/°), *t*(18) = 7.70, *p* < 0.001, *d* = 1.77). Furthermore, at an individual level, 18 out of 19 and 15 out of 19 participants in the VMR and the control group showed significantly positive slopes (*p* < 0.05). Thus, as instructed, participants implemented a parametric rotation as their strategy. We also obtained the intercepts (sFig. 1b, right): VMR (810.7 ± 51.17 ms vs. 709.6 ± 35.42 ms) and control reach (786.5 ± 26.96 ms vs. 690.1 ± 15.84 ms).

Then, we compared their improvement of the slopes (i.e., MR-rate, Fig. 2c, left) and intercepts (Fig. 2c, right) after visuomotor training by calculating a performance difference between the pre- and post-tests. In Fig. 2c, a larger positive slope and intercept reduction indicate a larger improvement in the rotational and non-rotational processes.

As depicted in Fig. 2c (left), we first confirmed that the VMR group showed significant MR-rate improvement after visuomotor training (*t*(17) = 3.94, *p* = 0.001, *d* = 0.93) but not for the control reach group (*t*(18) = 0.51, *p* > 0.250). Consequently, the VMR group showed a significantly larger MR-rate reduction than the control reach group (*t*(35) = 3.18, *p* = 0.003, *d* = 1.05). In other words, after VMR training, participants significantly improved the efficiency of rotational transformation. For other processing components, which are not associated with spatial transformation, both groups showed similar intercept improvement (*t*(36) = 0.17, *p* > 0.250), as shown in Fig. 2c (right). Therefore, we observed that VMR training led to a specific additional improvement in the mental rotation speed compared to the reach training.

In addition, we also looked into changes at the individual level after the visuomotor training session. We observed that while participants who had less mental rotation efficiency (larger MR rate) in the pre-test benefitted more (larger MR-rate reduction) from VMR training (slope = 0.31, *R*^*2*^ = 0.36, *p* = 0.011), there was no modulation in the control reach group (slope = -0.04, *R*^*2*^ = 0.01, *p* > 0.250) (Fig. 2d, left). This result confirms that only VMR training specifically facilitated the mental rotational process. In contrast, we observed that in both groups, individuals with slower RTs in the pre-test reduced their RTs more after the visuomotor training session (Fig. 2d, right), suggesting a common practice and motor training effect for other processes unrelated to rotation transformation (VMR: slope = 0.45, *R*^*2*^ = 0.86, *p* < 0.001; Reach: slope = 0.60, *R*^*2*^ = 0.66, *p* < 0.001).

By analyzing the motor performance of the two groups in the visuomotor washout session, we confirmed that participants maintained their motor learning until the end of the mental rotation post-test. In the VMR group, the mean reach error of the first trial block in visuomotor washout was -25.77° ± 1.78° s.e. (Fig. 2a: red), demonstrating a strong after-effect of VMR adaptation and indicating that participants still maintained partial adaptation to the 45° tilted visual feedback until the end of the mental rotation post-test. In the control reach group, the reach error of the first trial block was 0.30° ± 0.47° s.e. (Fig. 2a: blue), which was equivalent to the reach error of the last trial block (− 0.17° ± 0.40° s.e.) of the visuomotor training session (*t*(18) = 1.00, *p* > 0.025). This indicates that the control reach group maintained the same performance as in the visuomotor training session.

In sum, we observed that participants became faster overall in the mental rotation task after a short session of visuomotor rotation training. In contrast, training on the control motor task without the rotation operation resulted in a significantly weaker transfer to the mental rotation task. Importantly, we confirmed that such significant training effects by VMR were specifically led by improving the ability to transform spatial information, i.e., rotation per se, rather than other rotation-unrelated processes embedded in the mental rotation task. Thus, Experiment 1 uncovered that VMR training could improve mental rotation speed in accordance with the *domain-general* hypothesis.

### Experiment 2: mental rotation training improves visuomotor rotation

In Experiment 2, we reversely examined whether mental rotation training enhances visuomotor rotation while comparing it to other cognitive tasks without a rotation judgment. Such a bidirectional transfer between VMR and mental rotation provides strong converging evidence supporting a common *domain-general* operation. However, although we observed transfer from visuomotor rotation training to mental rotation in Experiment 1, if we do not observe the transfer in the opposite direction, it could indicate that rotation transfer is unidirectional from the motor to cognition, or alternatively, visuomotor rotation requires more complex movement control beyond a rotational operation, and mental rotation training is not sufficient to modify the adaptive performance required for visuomotor rotation.

Here, all participants performed four sessions: pre-test of VMR, visuomotor washout, visual training, and post-test of VMR. During the visual training session, participants were randomly assigned to perform the mental rotation or the control color-discrimination task (see “Methods: Experiment 2Tasks and Procedures” for details). The two VMR sessions and visuomotor washout were identical for the two visual training groups. The washout session was designed to remove the adaptation to the 45° tilted visual feedback and brought back visuomotor performance at the baseline.

We confirmed that only the mental rotation task but not the control color-discrimination task involved mental rotation processes (sFig. 2a in Supplementary material). In the mental rotation group, the RTs increased linearly with the rotation angles, suggesting that participants mentally rotated the letters from the rotation angle to the upright. However, the RTs of the control color-discrimination task were the same across all letter angles, indicating that the task did not involve the mental rotation process. We performed a linear regression on the averaged RT across the absolute letter-rotation angles regardless of the rotation directions (clockwise vs. counterclockwise) for the mental rotation and control color-discrimination group, respectively. The linear regression slope was significantly different from zero in the mental rotation group (slope = 1.65, *R*^2^ = 0.12, *p* < 0.001) but not in the control color-discrimination group (slope = -0.04, *R*^2^ < 0.001, *p* > 0.025). The accuracy of the mental rotation (90% ± 1.84% s.e.) and the control color-discrimination (91.54% ± 1.09% s.e.) task did not show a significant difference (*t*(34) = 0.72, *p* > 0.25).

**Figure 3:**
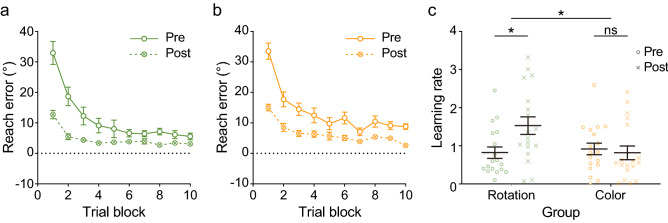
Results of Experiment 2. Reach error of VMR for the mental rotation group (a) and control color-discrimination group (**b**) in trial blocks 1–10 in the VMR pre-test and post-test. The pattern of lines and dots indicates test sessions (solid line/clear dot = pre-test, dotted line/crossed dot = post-test). (**c**) Learning rate (LR) of VMR tasks. Markers represent individual participants in the pre- (circle) and post-test (cross). Black horizontal lines represent the mean with standard errors of the mean in the corresponding group. In (**a**)–(**c**), the colors of the dots and lines correspond to visual training groups (green = mental rotation, orange = control color-discrimination). Error bars indicate standard errors of the mean. An asterisk indicates significant difference between results for the two training groups (*p < 0.05).

Of interest was whether VMR performance is differentially affected by the two visual training tasks. Based on our previous work, the learning process of visuomotor rotation mainly happens in the first ten trial blocks. We first calculated the reach errors of this period for the mental rotation group (Fig. 3a) and the control color-discrimination group (Fig. 3b) in the pre-and post-test sessions. Then we calculated the learning rate (LR) based on the reach errors in the pre-and post-test sessions (Fig. 3c) and the LR gain as an LR difference between the two test sessions. A larger LR gain indicates a larger VMR improvement. The mental rotation group showed a significantly larger LR gain than the control color-discrimination group.

A two-way repeated measures ANOVA with factors group and test session showed a significant interaction effect (*F*(1, 34) = 4.26, *p* = 0.047, η^*2*^ = 0.06), but no main effect of group (*F*(1, 34) = 2.40, *p* = 0.131) or test session (*F*(1, 34) = 3.54, *p* = 0.069). An independent t-test of LR gain showed that the mental rotation group had a significantly higher LR gain than the control color-discrimination group (*t*(34) = 2.07, *p* < 0.05, *d* = 0.69). Post hoc paired t-tests showed that while the LR in the pre-test session between the two groups was not significantly different (*t*(68) = 0.38, *p* > 0.250), only mental rotation group (*t*(34) = 2.56, *p* = 0.015, *d* = 0.56) but not the control color-discrimination group (*t*(34) = 0.37, *p* > 0.250) had LR improvement.

To get converging evidence, we further compared savings in reach error between VMR pre-and post-test for the two groups. Savings were calculated as the reach error difference between trial blocks 1–5 in the pre-and post-test. Larger savings indicate larger VMR improvement. The mental rotation (5.47° ± 1.69° s.e.) group showed significantly larger savings than the control color-discrimination (0.71° ± 1.21° s.e.) group (*t*(34) = 2.29, *p* < 0.03, *d* = 0.76) (sFig. 2b). Morehead et al.^58^ demonstrated that savings are likely to reflect the explicit processes. This pattern might indicate that participants improved their cognitive strategy to transform spatial information after the mental rotation training.

We also compared visuomotor washout performance across the two groups to ensure that the better VMR post-test performance in the mental rotation group was not induced by a difference in the visuomotor washout effect. We confirmed that the washout reach error between the mental rotation (− 9.96° ± 1.15° s.e.) and the control color-discrimination (− 11.9° ± 1.28° s.e.) groups were not significantly different (*t*(34) = 1.13, *p* > 0.250) (sFig. 2c).

In sum, we observed that after a short mental rotation training, participants became faster in visuomotor adaptation relative to the pre-training session, whereas training on the control color-discrimination task without the rotation operation did not facilitate the learning rate of visuomotor rotation. Such selective VMR improvement by mental rotation training provides strong converging evidence supporting the *domain-general rotational operation* hypothesis along with the results of Experiment 1.

## Discussion

In the present study, in the mental rotation tasks, participants rotated the internal representations of tilted visual stimuli^59^. Similarly, in the VMR task, participants learned how to rotate the internal representation of tilted visual feedback to determine the appropriate direction of hand movements^60^. Notably, we provided a direct experimental link between visuomotor and mental rotations beyond correlations. Specifically, in Experiment 1, we showed that training on visuomotor rotation adaptation resulted in significantly larger improvement in a mental rotation task than training on the reach task without a rotational component. In Experiment 2, we reversely demonstrated that the learning rate of visuomotor adaptation was improved after training on a mental rotation task but not after a control task that did not require a rotation judgment. Therefore, our results showing a bidirectional transfer are consistent with the notion that visuomotor rotation and mental rotation depend on a common rotation operation on spatially mapped representations of stimuli.

Our results are largely consistent with a meta-analysis of neuroimaging studies on mental rotation^39,52^ and visuomotor adaptation^61,62^. There is a robust involvement of dorsal frontoparietal regions such as intraparietal sulcus and superior parietal lobule areas. These processes are likely to track the allocation of attention and implementation of spatial maps, contributing to visuospatial representation transformation. In particular, the right posterior parietal cortex is also involved in mental spatial transformations based on coordinate spatial processing^63–65^. In addition, the supplementary motor area (SMA) and the pre-SMA were consistently activated. The role of SMA and pre-SMA is postulated in motor simulation and transforming spatial representations, which likely involves the sequential integration of elements into high-order representations. Pre-SMA projects to M1 and the spinal cord, placing it in a good position to play a role in motor control and simulation. Bilateral inferior frontal gyri opercularis and triangularis (BA 44 and 45), which were recently associated with motor control, imitation, and spatial maps, were also found active. These areas are consistent with prior studies of VMR adaptation^61,62^. Furthermore, Butcher et al.^66^ demonstrated that the dysfunction of the cerebellum, which showed significant brain activation during mental rotation tasks^67,68^, resulted in impairment in sensory prediction error-based learning and an explicit mental strategy such as aiming during visuomotor rotation.

Our observed bidirectional transfer can be understood together in the context of ‘embodied cognition’ research, which gives more credence to the motor system in playing a critical role in higher-level perceptual and cognitive functions^69–71^. The tenet is that perceptual and cognitive processes are grounded in bodily interactions with the environment^71–74^. While transfer from perception to action is well documented, for instance, in observational learning, transfer from action to perception has received much less attention. However, as we showed in Experiment 1, empirical evidence supports that self-generated action and action experience can modulate perception and cognition^28,75–77^. For example, blindfolded participants learned a novel coordinated upper-body movement based only on verbal and haptic feedback. The learned movement matched one of the visual test patterns, of which visual recognition was tested before and after the motor training session. Despite the absence of visual stimulation during training, participants displayed selective improvement in the visual recognition performance for the learned movement. This result demonstrated that the plasticity of visual recognition led by motor learning without visual learning supports a direct link between visual perception and motor systems^78^.

Our mental-sensorimotor workspace transfer is also compatible with several learning theories. For instance, the ideomotor theory suggests that actions are represented by their associated perceived effects: to generate an action, the observer can simply activate that internal representation of those effects, and the action will occur without any additional effort. A modern extension of the ideomotor theory is the theory of event coding (TEC)^79,80^. According to this framework, the final stages of perception and the initial stages of action control share a domain of coding where planned actions are represented in the same format as perceived events. One of the implications of this approach is that, under appropriate conditions, perceived environmental events can induce certain actions by way of similarity or feature overlap.

Alternatively, the symbolic learning theory^81^ postulates that mental practice, a training method by which an imagined motor act is mentally rehearsed multiple times without real movement execution^30,82^, leads to the rehearsal of the cognitive components of a motor task. According to this theory, training in mental rotation in Experiment 2 might induce a cognitive rehearsal of the rotational movement and, in turn, facilitate visuomotor rotation. Previous studies have also demonstrated that mental practice can improve motor skill performance^29,83,84^. However, since the imagined movement associated with mental rotation on tilted objects is not the same as the rotational movement involved in VMR, further study is needed to fully understand how and whether mental practice on rotating tilted letters could benefit the VMR learning process.

To conclude, by combining methods and insights from cognitive psychology and motor control, our work demonstrated remarkable reciprocal plasticity achieved through the two widely different tasks. This newly observed reciprocal enhancement between the cognitive and motor processes, which could not be explained by a unidirectional sequence of information processing^1–4^, highlights that human actions are not merely the outcomes of internal mental functions. However, they can influence cognitive processes. By demonstrating intertwined operations in mental and sensorimotor workspaces, the present work represents a substantive departure from the status quo and urges future studies to shift focus to a new interactive framework between cognitive and sensorimotor domains. Understanding their interrelation with an integrated approach will significantly enhance our understanding of the dynamic interdependence between cognitive and sensorimotor mechanisms.

## Methods

### Participants

In Experiment 1, 38 right-handed Brown University students participated in the one-hour study for course credit or monetary compensation, reported normal or corrected-to-normal color vision, and were naïve to the aims of the experiment. Nineteen participants (15 females, mean age 21.26 years) were included in the visuomotor rotation group, and 19 participants (19 females, mean age 19.58 years) were included in the reach group.

In Experiment 2, 36 right-handed Brown University students participated in the one-hour study for course credit or monetary compensation. Each reported normal or corrected-to-normal color vision and was naïve to the aims of the experiment. Eighteen participants (9 females, mean age 20.78 years) were included in the mental rotation group, and 18 participants (12 females, mean age 19.33 years) were included in the control color group. The sample size (18 per group) was determined based on the effect size needed to achieve 80% statistical power (η^2^ = 0.12, *n* = 15), as determined with a one-way ANOVA on preliminary data.

All experimental protocols were approved by the Brown University Institutional Review Board (IRB) in accordance with the Code of Ethics of the World Medical Association (Declaration of Helsinki) for experiments involving humans. All research was performed in accordance with the approved IRB guidelines. Informed consent was obtained from all the participants.

### Apparatus

All experiments were conducted in a dimly illuminated room. Participants sat in front of a 21.5-inch Apple iMac computer, and all stimuli were presented with a 60 Hz refresh rate and 1920 × 1080 pixel resolution. In the visuomotor rotation and reach tasks, participants held a stylus pen with their right hand and moved the pen across the surface of a touch screen (Magic Touch; Keytec, Garland, TX), which lay flat on a table and aligned with the midlines of participants and the computer screen. Participants could not see their hands during reach movements. The stylus movement controlled a corresponding cursor on the computer screen. Stimulus presentation and recording of cursor displacement were conducted using custom software designed with MATLAB (2011a, Mathworks) and Psychtoolbox^85^.

### Experiment 1

#### Tasks

*Mental rotation task.* Four asymmetric upper-case letters (F, G, J, R) and their horizontally flipped (mirrored) images were used as stimuli for a total of eight characters (font: Courier New, height: 2.82 cm, color: white). Each stimulus was rotated in 45° increments from − 135° (counterclockwise from upright) to 135° (clockwise from upright), for a total of eight angles (Fig. 1b). On each trial, a white fixation dot (0.24 cm diameter) appeared at the center of the screen (black) and stayed for 1000 ms. Then, the stimuli were presented at the same location as the fixation dot. Participants were instructed to mentally rotate the letters to the upright position and determine whether each stimulus was a normal letter or a mirrored image by pressing a button with their right hand (“1” for normal, “2” for mirror) and were required to respond as accurately and as quickly as possible. The stimuli stayed on the screen until a button was pressed, but no longer than 5000 ms. Distinct auditory feedback was presented to inform the accuracy of button-press responses. One block consisted of 64 trials (8 stimuli × 8 angles) in random order.

*Visuomotor rotation (VMR) task* (Fig. 1c, red border)*.* Participants were instructed to move a cursor on the screen from the starting base in the center of the screen toward a reach target. The starting base was a white circle with a diameter of 1 cm, and the reach target was a solid white dot with a diameter of 1 cm located 5.5 cm from the starting base. At the beginning of each trial, the initial location of the cursor was outside the starting base. Participants moved the cursor into the starting base to trigger the appearance of the target, which stayed on the screen for 1500 ms. Each target direction was located in 45° increments from 0° (12 o'clock) to 315°for a total of eight possible target locations.

Participants were asked to move the cursor toward the target as fast and as linear as possible and move it back to the starting base immediately after they reached the target. There were two types of trials. In rotation trials, the cursor direction (solid line) was rotated 45° clockwise (CW) from the hand trajectory (dotted line). This perturbation creates movement errors, forcing the brain to learn or update new sensory-motor relationships to reestablish appropriate motor control. In no-rotation trials, the cursor direction (solid line) normally followed the hand trajectory (dotted line). This task included (1) one practice block (24 no-rotation trials), (2) one baseline block (80 no-rotation trials), measuring inherent bias in the reaching movement toward each target, and (3) four training blocks (4 × 80 rotation trials, ten trials for each of the eight reach targets). Eight target locations were presented randomly within every eight trials in each block.

*Control reach task* (Fig. 1c, blue border)*.* Everything was the same as the VMR task, except the cursor direction followed the hand trajectory normally without rotation (no-rotation trials). This task was composed of (1) one practice block (24 no-rotation trials), (2) one baseline block (80 no-rotation trials), and (3) four reach blocks (4 × 80 no-rotation trials, ten trials for each of the eight reach targets). Eight target locations were presented randomly within every eight trials in each block.

#### Procedures

All participants performed four sessions: (1) the pre-test of the mental rotation task, (2) visuomotor training, (3) the post-test of the mental rotation task, and (4) visuomotor washout (Fig. 1a). After one practice block of the mental rotation task (Fig. 1b), all participants performed the pre-test of mental rotation task (4 blocks) to assess their baseline performance. During the visuomotor training session, participants were randomly assigned to perform the visuomotor rotation (VMR) task or the control reach task, followed by the post-test of the mental rotation session (4 blocks). The visuomotor washout session was designed to ensure that the visuomotor training effects were maintained during the post-test of mental rotation. The VMR and reach groups performed their baseline block (80 no-rotation trials).

#### Data analysis

Performance for the mental rotation task was measured by reaction time, the duration between the letter onset and button response, of all correct trials in each test session. The letter rotation angles were calculated as the shortest rotated angles between the stimuli and their upright collapsing clockwise and counterclockwise directions. The linear regression was performed as a function of letter rotation angles (sFig. 1a). Data analysis procedures for VMR and control reach tasks followed our previous studies^10,24,26^. We differentiated the position of the cursor to obtain tangential velocity. The onset and offset of the movement were defined as when the cursor speed exceeded and fell below 5% of peak velocity, respectively. Reach error was defined as the angle difference between the line joining the starting base to the target and the line joining the cursor locations at movement onset and peak velocity. Reach error was averaged across eight successive trials as a trial block covering all target locations.

We used MATLAB (2020a, Mathworks) and Prism 9 for macOS (GraphPad Software, La Jolla California USA, www.graphpad.com) to perform all data processing and statistical analysis. Outliers were detected and excluded before performing statistics by the combined robust regression-and-outlier-removal (ROUT) method^86^. We used parametric ANOVA and mixed-effects analysis to analyze the data. If multiple post hoc comparisons followed, we used Šidák correction^87^ for comparisons between multiple treatment groups. Greenhouse–Geisser correction^88^ was applied when the assumption of sphericity was violated. All error bars represented the standard error of the mean (SEM). The effect size of ANOVAs and t-tests were measured by eta-squared (η^2^) and Cohen’s *d*, respectively. According to Cohen^89^, η^*2*^s of 0.01, 0.06, and 0.14 and Cohen’s *d*s of 0.20, 0.50, and 0.80 are considered small, medium, and large effect sizes, respectively.

### Experiment 2

#### Tasks

The same mental rotation and VMR tasks as in Experiment 1 were used except for the following modifications. In the mental rotation task, in each trial, letter stimuli were presented in one of two colors: pink (RGB: 255, 240, 255) and purple (RGB: 240, 255, 255). The stimulus color was randomized within each block with an equal number. We also added one new control task by modifying a task instruction of the mental rotation task with the same display. The control color-discrimination task required participants to indicate whether the stimulus was pink or purple by pressing a button with their right hand (“1” for pink, “2” for purple) and were required to respond as accurately and as quickly as possible regardless of its rotation angle.

#### Procedures

To examine a reverse transfer from mental rotation training to VMR, all participants perform the following four sessions: (1) pre-test of VMR task, (2) visuomotor washout of VMR task, (3) visual training, and 4) post-test of VMR. The pre-test VMR sessions were identical to the VMR task of the VMR group in Exp. 1, including one practice block, one baseline block, and four training blocks. The post-test VMR sessions include four training blocks. The washout session (80 no-rotation trials) was designed to remove the adaptation to the 45° tilted visual feedback and brought back reach to the baseline level. During the visual training session, participants were randomly assigned to perform the mental rotation or the control color discrimination task.

#### Data analysis

In addition to the analyses used in Experiment 1, we calculated the learning rate (LR) in the VMR tasks to indicate the learning performance. The LR is quantified based on the reach errors in the first block of each session, using Eq. (1).

1 $$y= \mathrm{a}+\mathrm{b}*{\mathrm{e}}^{\left(\mathrm{c}* -\mathrm{x}\right)},$$

where y is the averaged reach error of each trial block in the first block; a is the level of performance at the end of the block; b is the adjusted initial performance; c is the learning rate, representing the rate of visuomotor adaptation; x is the trial block index from 1 to 10, representing the time course of the visuomotor adaptation. The initial values of a and b for the model-fitting are the reach error in trial block ten and the absolute reach error difference between trial block 1 and 10, respectively. The learning rate c was fitted as a free parameter larger than 0.

In addition, savings were calculated as the reach error difference between trial blocks 1–5 in the pre-and post-test. Larger savings indicate larger VMR improvement (sFig. 2b). The washout reach error was calculated as the mean reach error of the washout session for each group, respectively (sFig. 2c). To control an individual difference led by the washout session, we adjusted post-test performance by subtracting an error of that last washout trial block within each participant.

## Supplementary Information

Supplementary Figures.

## Data Availability

The data that support the findings of this study are available from the corresponding author upon request.
