# Supplementary material for: Reciprocal facilitation between mental and visuomotor rotations
Source: Sci Rep. 2023 Jan 16;13:825. doi: 10.1038/s41598-022-26397-3 (PMC9842739; doi:10.1038/s41598-022-26397-3)
Supplement: Supplementary file 1 — Supplementary Figures. [file 41598_2022_26397_MOESM1_ESM.pdf]

Supplemental material

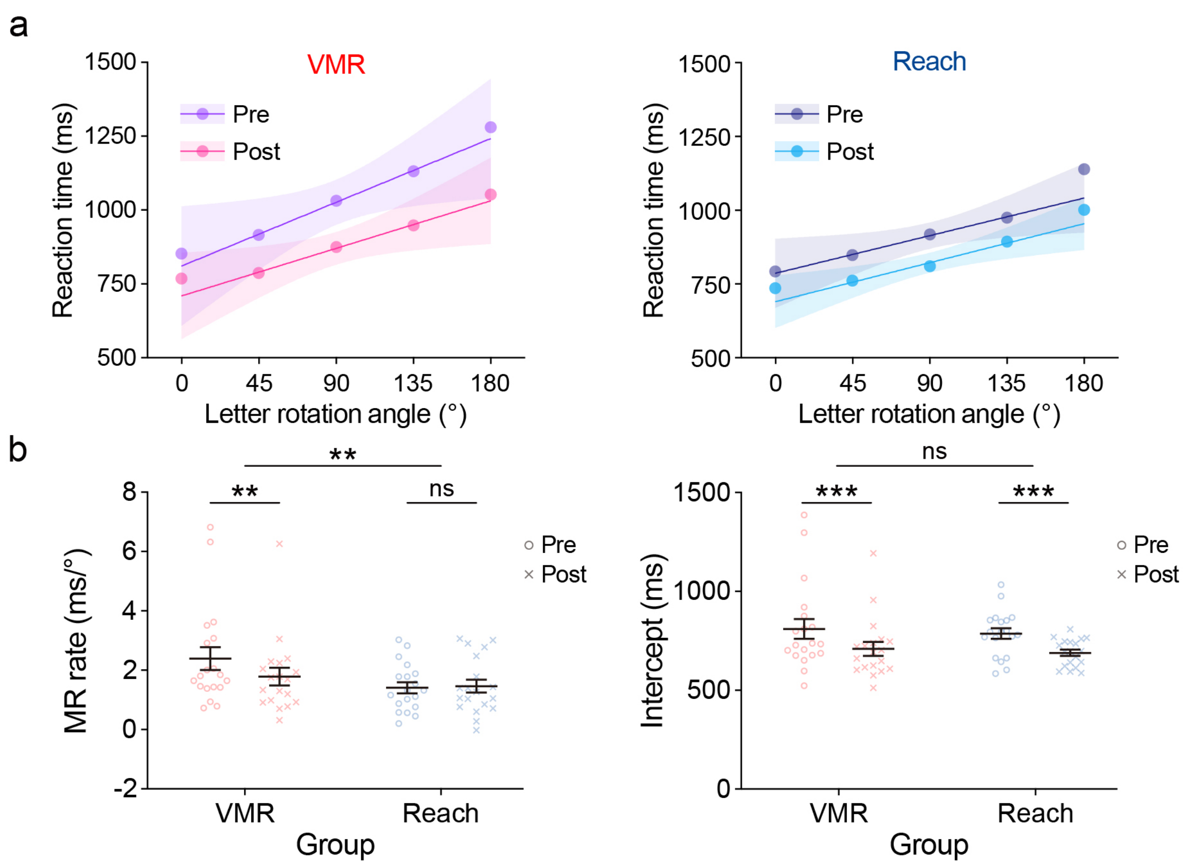

sFig. 1. (**a**) RTs of mental rotation in VMR (left) and control reach group (right) as a function of the letter rotation angle. The linear regression line was averaged based on regression lines between letter rotation angles 45 - 135 degrees across all participants in each group during the pre- and post-test separately The colors of the markers, lines, and ribbons correspond to visuomotor training groups and test sessions (VMR-pre (purple): y =2.40*x + 810, R^2 = .09, p = .025; VMR-post (pink): y =1.79*x + 709, R^2 = .09, p = .020; control reach-pre (dark blue) y =1.42*x + 786, R^2 = .09, p = .022; control reach-post (light blue): y =1.47*x + 690, R^2 = .16, p = .002). Each circle represents the mean RT at each letter rotation angle. Ribbons indicate a 95% confidence interval. (**b**) Mental rotation (MR) rate (left) and the intercept (right) of mental rotation tasks in the VMR and reach groups. Markers represent individual participants in the pre- (circle) and post-test (cross). Black horizontal lines represent the mean with standard errors of the mean in the corresponding group. The colors of the markers and lines correspond to visuomotor training groups, as depicted in Figure 1c (red: VMR and blue: control reach). Error bars indicate standard errors of the mean. An asterisk indicates significant difference between results for the two training groups (**p < .01, ***p < .001).

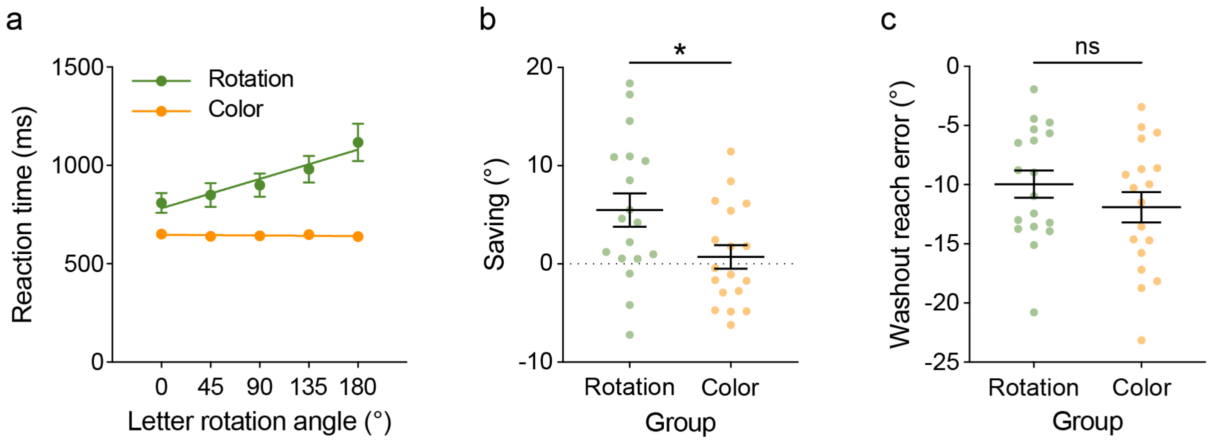

sFig. 2. (**a**) RTs of mental rotation and control color-discrimination tasks. The linear regression line for each group was averaged based on regression lines across all participants. (**b**) Savings of mental rotation and control color-discrimination tasks. (**c**) Washout reach error of mental rotation and control color-discrimination tasks. In (**b**) and (**c**), dots represent individual participants in the corresponding group. Black horizontal lines represent the mean with standard errors of the mean in the corresponding group. In (**a**) to (**c**), the colors of the dots and lines correspond to visual training groups (green = mental rotation, orange = control color-discrimination). In all figures, error bars indicate standard errors of the mean. An asterisk indicates a significant difference between the two training groups (*$p < .05$).
